# Characterization of Clostridium Baratii Type F Strains Responsible for an Outbreak of Botulism Linked to Beef Meat Consumption in France

**DOI:** 10.1371/currents.outbreaks.6ed2fe754b58a5c42d0c33d586ffc606

**Published:** 2017-02-01

**Authors:** Christelle Mazuet, Christine Legeay, Jean Sautereau, Christiane Bouchier, Alexis Criscuolo, Philippe Bouvet, Hélène Trehard, Nathalie Jourdan Da Silva, Michel Popoff

**Affiliations:** Institut Pasteur, Bactéries Anaérobies et Botulisme, Paris, France; Institut Pasteur, Bactéries Anaérobies et Botulisme, Paris, France; Institut Pasteur, Bactéries Anaérobies et Botulisme, Paris, France; Institut Pasteur, Plateforme Génomique-Pôle Biomics, Paris, France; Institut Pasteur, Hub Bioinformatique et Biostatistique, CNRS, Paris, France; Institut Pasteur, Bactéries Anaérobies et Botulisme, Paris, France; French Institute for Public Health Surveillance, InVS, Coordination of Alerts and Regions, Regional Office in Rhone-Alpes, Lyon, France; French Institute for Public Health Surveillance, InVS, Infectious Disease, Saint Maurice, France; Institut Pasteur, Bactéries Anaérobies et Botulisme, Paris, France

## Abstract

**Introduction:**

A second botulism outbreak due to Clostridium baratii occurred in France in August 2015 and included three patients who had their meal in a restaurant the same day. We report the characterization of C. baratii isolates including whole genome sequencing (WGS).

**Methods:**

Four C. baratii isolates collected in August 2015 from the outbreak 2 were analysed for toxin production and typing as well as for genetic characterization. WGS was done using using the NEBNext Ultra DNA Library Prep kit for Illumina (New England Biolabs) and sequenced on MiSeq machine (Illumina) in paired-end reads of 250 bases. The phylogenetic tree was generated based on the UPGMA method with genetic distances computed by using the Kimura two-parameter model. Evolutionary analyses were conducted in Bionumerics (V.6.6 Applied Maths).

**Results:**

Three C. baratii isolates for patient's stools and one isolate from meat produced botulinum neurotoxin (BoNT) type F and retained a bont/F7 gene in OrfX cluster. All isolates were identical according to the WGS. However, phylogeny of the core genome showed that the four C. baratii strains were distantly related to that of the previous C. baratii outbreak in France in 2014 and from the other C. baratii strains reported in databanks.

**Discussion:**

The fact that the strains isolated from the patients and meat samples were genetically identical supports that the meat used for the Bolognese sauce was responsible for this second botulism outbreak in France. These isolates were unrelated to that from the first C. baratii outbreak in France in 2014 indicating a distinct source of contamination. WGS provided robust determination of genetic relatedness and information regarding BoNT typing and toxin gene locus genomic localization.

## INTRODUCTION

Botulism is a rare but severe neurological disorder characterized by a flaccid paralysis that, in the most severe forms, leads to respiratory failure. The disease is due to botulinum neurotoxins (BoNTs) which are produced by diverse anaerobic spore-forming bacteria from the genus *Clostridium* including *Clostridium botulinum*^2^ (groups I to III), *Clostridium argentinense*, *Clostridium baratii*, and *Clostridium butyricum *1^,^^31^^,^^2^. Food-borne botulism is the main form of botulism in France. Between 20 and 40 cases of human botulism are reported every year. Homemade ham or ham processed by small-scale enterprises is the most frequent source of human type B botulism in France 3^,^^4^^,^^5^. However, since 2005 an increased number of other types of botulism such as type A were observed due to changes in food habits, as well as in food production and distribution including increased development of commercial minimally-heated, chilled foods, and imported products 5. Type F botulism was first reported in Denmark in 1958 and was due to a homemade liver paste contaminated with a novel *C. botulinum* type called type F 6. Most cases of human botulism are caused by BoNT types A, B or E, and more rarely by type F. For example, botulism type F represented 1% of botulism cases in the US within the period 1981-2002 7. In 1980, BoNT/F was recognized to be produced not only by *C. botulinum* but also by a distinct *Clostridium* species called *C. baratii* 8 . Since this period, *C. baratii* is the most frequent cause of type F botulism. In the US from 1981 to 2002, C. baratii was isolated from 9 of the 13 cases of type F botulism, and *C. botulinum* F was not detected in any 7. *C. baratii* type F (referred to as F7) 9 is mainly involved in infant botulism and in adults with botulism by intestinal colonization 7^,^^10^^,^^11^^,^^12^^,^^13^. Only a few type F food borne botulism outbreaks were reported 7^,^^14^. Here, we report the characterization of *C. baratii* strains from the second documented food-borne botulism outbreak in France 15 and the genetic relationship between strains of the two French outbreaks based on core genome analysis.

## MATERIALS AND METHODS

**Ethics Statement**

All experiments were performed in accordance with the French and European Community guidelines for laboratory animal handling (agreement of laboratory animal use n° 2013-0116/02026.02 and n°2013-0117/02025.02).

**Toxin Detection**

Toxin detection and titration in biological samples or in culture supernatants were performed by the mouse bioassay with specific neutralizing antibodies from the National Reference Centre (NRC) of Anaerobic bacteria and Botulism (Institut Pasteur, Paris) and/or National Institute for Biological Standards and Control (London, UK). Neutralizing polyclonal antibodies were prepared at the NRC by immunization of rabbits with recombinant half C-terminal of BoNT heavy chain (Hc domain) (3 to 5 subcutaneous injections of 400 μg Hc in 1 ml PBS with 1 ml Freund adjuvant) (data not shown). One ml of patient's serum or half ml of ten-fold serial dilutions of stool or food samples in 50 mM phosphate buffer (pH 6.5) containing 1% gelatin were injected intraperitoneally into Swiss mice weighing 20-22 g (Charles River).

**Cultures**

Enrichment cultures of stool or food samples were performed in fortified cooked meat medium (FCMM, Difco) at 37°C in anaerobic conditions and *C. baratii* was isolated on sheep blood FCMM agar as previously described 16. Stool and food samples were not heat or ethanol treated prior to enrichment cultures. The strains were grown in TGY (Trypticase, yeast extract, glucose) 17.

**DNA Preparation, PCR detection**

DNA extraction from stool samples was performed with either QIAamp DNA Stool kit (Qiagen) for stool specimens or Power Food microbial DNA extraction kit (MO BIO Laboratories) for food samples according to the manufacturer's recommendations. Detection of *C. botulinum* in biological samples was performed by SYBR-green real-time PCR with specific primers as previously described 18. A pair of primers was designed to cover bont/F1 and bont/F7 genes (P2308 /P2309). These primers were selected using Primer3 program (v.0.4.0) in a conserved sequence of BoNT / F1 and F7 (préciser les positions?). To provide evidence that thesis newly designed primers were specific to bont/F1 and / F7 genes, PCR was performed with the DNA of the strain NCTC10281 (bont / F1) and ATCC43756 (bont / F7). The presence of the amplification product was confirmed on agarose gel. The sequencing of the amplification products (Eurofins / MWG) confirmed the specificity of the primers (data not shown) 18.

Total DNA was isolated from *C. baratii* strains as described 18.

**Whole genome sequencing**

WGS libraries were performed using the NEBNext Ultra DNA Library Prep kit for Illumina (New England Biolabs) and sequenced on MiSeq machine (Illumina) in paired-end reads of 250 bases. Sequence files were generated using Illumina Analysis Pipeline version 1.8 (CASAVA). After quality filtering, reads were assembled using CLC software version 4 (CLC Bio).

**Bioinformatic analyses**

16s rRNA gene analyses were performed by BlastN.

Dendograms representing the phylogenetic relatedness of BoNT/F7 nucleotide sequences and deduced proteins were constructed using the UPGMA method. The genetic distances were computed by using the Kimura two-parameter model. Evolutionary analyses were conducted in Bionumerics (V.6.6 Applied Maths)

To compare the genomic environment of BoNT/F7 toxin clusters, the sequence between the AraC family transcriptional regulator and ABC transporter substrate-binding protein genes of *C. baratii* strain Sullivan was aligned with contigs carrying the BoNT/F7 operon of strains 796-15 and 771-14 using BlastN, and compared using Artemis Comparison Tool 22.

Maximum likelihood phylogenetic tree of seven *C. baratii* isolates was inferred from 2.51M recombination-purged aligned characters belonging to the core-genome induced by this taxonomic sampling.

## RESULTS

**Case report**

****In August 2015, an outbreak of botulism including three persons who had their meal on the same day at the same restaurant occurred in a city of the South of France 15. The three patients developed a severe botulism including quadriplegia and respiratory failure. They were hospitalized and required mechanical ventilation 15. The common food eaten by the three patients was pasta with Bolognese sauce which had been prepared one or two days before it was served. No leftover of the Bolognese sauce was available for investigation, but frozen and defrosted ground meat samples from the batch which had been used to prepare the sauce and which were stored in the restaurant at -20°C and +4°C, respectively, were collected.

**Botulinum** **toxin detection in biological and food samples**

Evidence for toxicity in serum samples of the three patients was provided by using the mouse bioassay (Table 1). The mice developed the characteristic signs of botulism, but the toxicity was not neutralized with specific antibodies against botulinum toxin (BoNT) from *C. botulinum* type A, B, or E. BoNT was detected at 40 and 2,000 MLD (mouse lethal doses)/g in the stools of two patients, respectively (Table 1), and BoNT/F gene was identified. Among 21 samples of food at risk of botulism, *C. baratii* was identified and isolated from the meat samples stored in the restaurant either frozen at -20°C or defrosted and preserved in refrigerator 15 (Table 1). However, BoNT was not detected in the meat samples.

**Table 1:** Table 1: Botulinum toxin (BoNT) and C. baratii investigation in biological and meat samples. a Toxicity in serum samples was not neutralized by anti-A, -B, and –E sera. Neutralization with anti-F was not tested due to insufficient sample volumes. b BoNT typing was performed by neutralization test with 0.5 ml of stool dilution containing 4 to 8 MLD (mouse lethal dose) and mixed with 50 μl of anti sera containing 0.25 international units (IU). No neutralization was obtained with anti-A, -B, and –E sera, and a partial neutralization (mouse death delayed of 24-48 h compared to control mice injected with samples without C. botulinum antisera) was observed with anti-F serum raised against C. botulinum F toxoid (50 μl antiserum containing 0.25 IU). c BoNT 1 MLD indicates that mice injected with 1 ml patient's serum developed characteristic botulism symptoms but did not die within 4 days of observation. d BoNT/F was identified in the FCMM enrichment culture. MLD, mouse lethal dose.

Patient/Food	Serum^a^ (BoNT MLD/ml)	Stool		
		BoNT^b^ MLD/g	bont/F gene PCR detection	C. baratii strain
n° 1	1-4	2,000	+	694-15
n° 2	< 1^c^	no detected^1^ < 10	+	693-15
n° 3	≥ 1	40	+	695-15
		Food		
Two frozen ground meat and defrosted ground meat samples		no detected < 6	+	796-15

***C. baratii* isolation and strain characterization**

**Figure 1:**
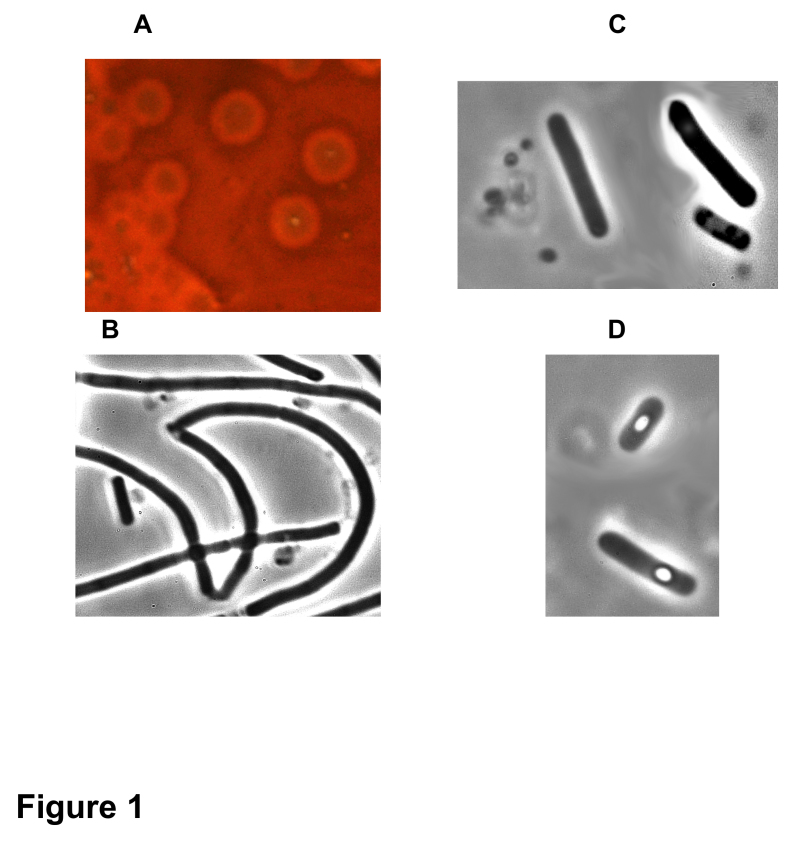
**Fig 1. Colonies and morphology of *C. baratii* 694-15.** (A) Colonies on sheep blood TGY agar surrounded by hemolysis halo. Phase contrast microscopy (magnification 1000x^31^) of culture in TGY broth in the exponential growth phase (B), stationary phase (C), and sporulation phase (D).

Four *C. baratii* F strains were isolated from stool and meat samples (Table 1). They showed the same morphological (Fig. 1) and bacteriological properties as well as antibiotic resistance profile (data not shown) as the two strains isolated in the previous *C. baratii* outbreak in France (outbreak 1) 16. All the strains grown in TGY medium produced BoNT as assayed in mouse test which was partially neutralized by anti-*C. botulinum* F serum, and to a lower extent by anti-E and not by anti-A or anti-B serum (Table 1.

Whole genome sequencing (WGS) of the four strains (693-15, 694-15, 695-15 and 796-15) of this outbreak was performed. The BlastN analysis of DNA sequences confirmed that 16S rRNA genes of the four isolated strains were 99% related to that of *C. baratii/C. sardiniensis* in databank, and showed the presence of only one bont/F7 locus associated with orfX genes.

**Figure 2:**
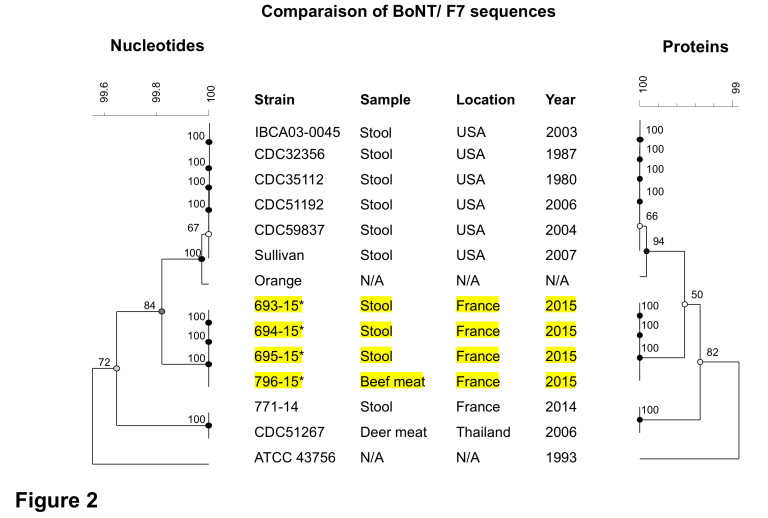
**Fig 2. Phylogenetic relatedness of BoNT/F7 nucleotide sequences and deduced proteins.** The dendograms were constructed using the UPGMA method. The scale bar indicates similarity values. The numbers next to each node indicate the bootstrap values (n=100). Strains noted * were isolated from the same botulism outbreak. Genbank accesion numbers are: IBCA03-0045, JX847735; CDC32356, GU213234; CDC35112, GU213233, CDC51192; GU213232; CDC59837, GU213231; Sullivan, CP006905; Orange, HM746655; CDC51267, GU213235; ATCC 43756, X68262.

A comparison of *bont*/F7 nucleotide and deduced protein sequences encoded by strains isolated from the outbreaks 1 and 2 16 in France completed with those available in public databases confirmed a high degree of homogeneity (at least 99% identity) of this neurotoxin (Figure 2). However, the subtype F7 is the most divergent compared to other F subtypes 19. BoNT/F7 encoded by the four strains of outbreak 2 were identical to each other and differed by 0.6% (7 amino acid substitutions) from that of the 771-14 strain from the outbreak 1 16, suggesting that the strains from the two outbreaks were different. Very interestingly, bont/F7 of 771-14 strain is 100% identical to that of the strain CDC 51267 isolated from deer meat associated with a botulism outbreak in Thailand in 2006 19 while all the 6 *C.baratii* strains isolated from individual botulism cases in USA between 1980 and 2007 20^,^^2^^,^^19^ encode the same BoNT/F7 sequence distinct from those of the strains of other countries (Fig. 2). All BoNT/F7 retain amino acids which are critical for their activity. Notably W312 and Y314 which are essential in the binding to the substrate, synaptobrevin2 21^,^^20^ are conserved in the *C. baratii* isolates from France as well as in C. baratii reference strains (Fig. 2). This suggests that the C. baratii strains including the French isolates share the same substrate specificity.

**Flanking regions of BoNT/F7 cluster**

**Figure 3:**
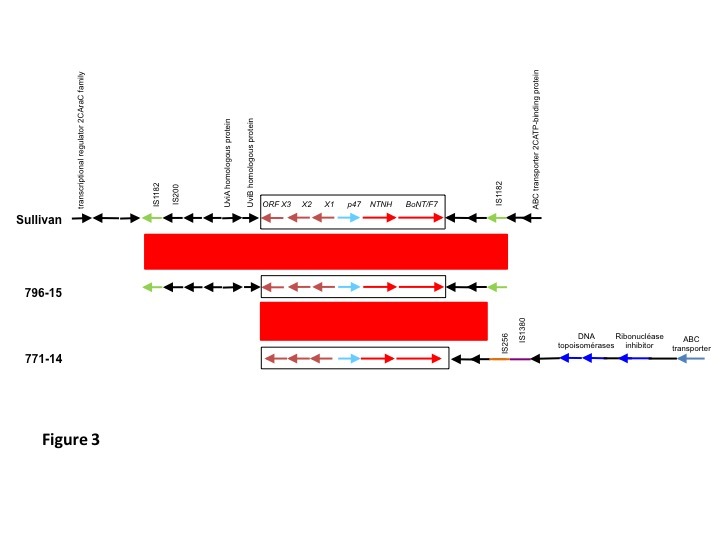
**Fig 3. Genomic environment of BoNT/F7 toxin cluster**The sequence between the AraC family transcriptional regulator and ABC transporter substrate-binding protein genes of C. baratii strain Sullivan was aligned with contigs carrying the BoNT/F7 operon of strains 796-15 and 771-14. The conserved regions (≥ 99% identity) are indicated in red.

It was previously shown that the *bont*/F7 cluster is flanked by two IS1182 copies 2. The genomic environment of the bont/F7 cluster was analyzed by comparing the flanking regions from AraC family transcriptional regulator to ABC transporter substrate-binding protein of *C. baratii* Sullivan with the contig carrying the bont/F7 operon of strains 796-15 and 771-14 (Figure 3). The ca. 20 kb regions were aligned using BlastN and compared using Artemis Comparison Tool 19. Interestingly, the *bont*/F7 cluster and flanking parts of the strain 796-15 (outbreak 2) were almost identical to that of strain Sullivan (99% identity), at least between the two intact direct repeats of IS1182. Upstream of the orfX3 gene, we found almost the same UviA-like putative sigma factor operon (99.6% and 100 % nucleic acid identity with *uviA* and *uviB*, respectively) as that described for the strain IBCA03-0045 20. In strain 771-14 (outbreak 1), the BoNT/F7 cluster was also almost identical to those of strains Sullivan and 796-15 (99% identity) but the insertion sequences IS256 and IS1380 instead of IS1182 lied downstream (Fig. 3). In addition, the flanking genes downstream from the *bont*/F7 cluster showed significant homology with DNA modifying genes such as topoisomerase from the *Clostridium perfringens* plasmid pCP-TS1 16. This suggests different integration events and that the *C. baratii* strains from the two outbreaks in France are different strains.

***C. baratii* core genomes**

**Figure 4:**
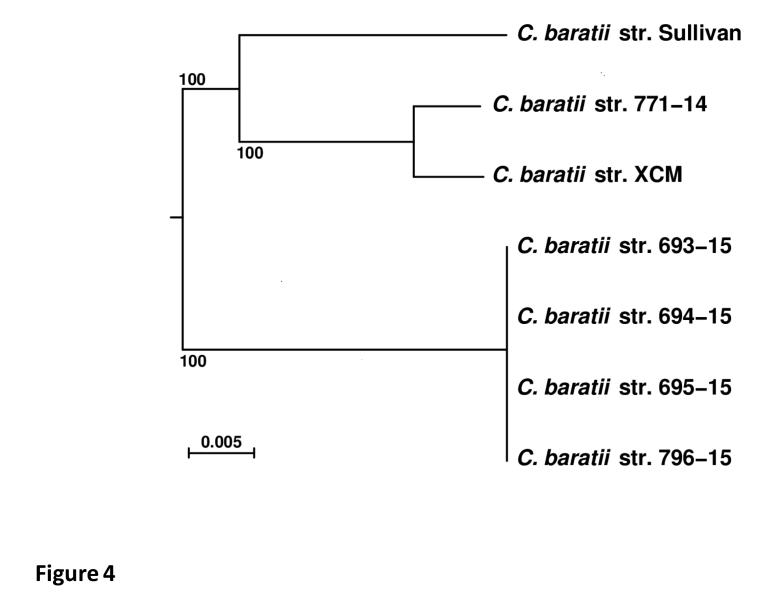
**Fig 4. Phylogenetic tree of seven *C. baratii* isolates. **Scale bar represents 0.005 nucleotide substitutions per character. Confidence supports at branches were estimated by a bootstrap procedure (500 replicates). Rooting was performed by using  the genome sequence of *C. perfringens* strain SM101 as an outgroup (not shown).

To further investigate the genetic relationship between these two strains, we constructed a core genome SNP phylogeny. The initial five sets of contigs (strains 771-14, 693-15, 694-15, 695-15 and 796-15) were  compared with two other publicly available C. baratii genome sequences, i.e. strains XCM  (NCBI accn. LGRR00000000) and Sullivan (NCBI accn. CP006905). These seven sets of genome sequences were processed with the Harvest suite 32 in order to infer a core-genome purged from any region having undergone likely horizontal evolutionary events (e.g. homologous recombination), leading to 2.51M aligned nucleotide characters. A phylogenetic tree was inferred from these aligned characters by PhyML 23^,^^24^ with evolutionary model GTR+G4+I (Figure 4). The core genomes confirm that the four strains from outbreak 2, isolated from patients samples (693-15, 694-15 and 695-15) and beef meat sample (796-15) were identical to each other and distantly related to the strain 771-14 from outbreak 1, as well as to the other *C. baratii* strains, Sullivan from the U.S., and XCM from China. The *C. baratii* strain 771-14 seemed to be more related to the Chinese strain isolated from soil in 2014. Interestingly, we did not find *bont*/F7 gene sequence on the contigs of the XCM strain suggesting that this *C. baratii* strain could have lost or never acquired this locus while the *orfX-bont*/F7 locus is possibly located on a plasmid in strain 771-14 16.

## 

## DISCUSSION

*C. baratii* producing BoNT/F was first identified in an infant botulism case from New Mexico (USA) in 1980 8. Then, several cases of *C. baratii* type F botulism have been reported in babies, mainly in the U.S. 25^,^^26^^,^^28^. *C. baratii* was also recognized as responsible for botulism in adults 14^,^^26^^,^^27^. However, food borne botulism outbreaks linked to *C. baratii* type F are rare, approximately 1.1 % of the botulism cases in the U.S. from 1981 to 2011 2^,^^7^. Most of the food borne botulism outbreaks linked to C. baratii F were reported in the U.S. One *C. baratii* outbreak was described in 5 adults in Spain in 2011 35. The first botulism outbreak due to *C. baratii* F identified in France was in November 2014 and includied a very severe case and a mild form of botulism 34. The second outbreak involved three persons who had their meal on the same day and in a same restaurant 15. Most of botulism cases in adults associated with C. baratii were attributed to botulism by intestinal colonization 7. Based on a short duration from consumption of the suspected food to symptom onset in the three patients (duration mean, 32 h) 15, the botulism form in the outbreak 2 is more likely a food-borne botulism than botulism by intestinal colonization.

The four *C. baratii* strains of the outbreak 2 were identical at the genetic level and were distantly related to the other *C. baratii* strains from the outbreak 1 in France and from those isolated in the U.S. and China indicating a distinct origin of the corresponding outbreaks (Fig. 4). The genetic diversity of *C. baratii* is still poorly understood since a limited number of strains has been sequenced and analysed until now. The *bont*/F7 locus shows genetic variations albeit to a lower extent than the whole genome (Fig. 2 and 4). Interestingly, the four *C. baratii* strains from the outbreak 2 retain conserved *bont*/F7 loci including the flanking IS1182 identical to that of the Sullivan strain, whereas the strain 771-14 from outbreak 1 shows a variant *bont*/F7 locus with distinct flanking regions (Fig. 3). However, the core genome of the four *C. baratii* strains of outbreak 2 is more divergent than that of 771-14 compared to the Sullivan strain (Fig. 4). This further supports horizontal *bont*/F7 gene locus transfer between strains with different genetic background. The *bont*/F7 locus insertion site in the chromosome is similar in 796-15 and Sullivan strains, whereas in strain 771-14 the botulinum locus has a distinct location, likely on plasmid (Fig. 3). The previously characterized bivalent strains Af and Bf also argue for horizontal gene transfer of the bont/F locus mediated by plasmids and recombination events in various *C. botulinum* strains, and probably between *C. botulinum* and *C. baratii* 29^,^^30^^,^^33^.

The contamination origin of the botulism outbreaks caused by *C. baratii* type F remains largely unknown. *C. baratii* F and BoNT/F were identified in a spaghetti sauce meat preparation responsible for a botulism outbreak in the U.S. in 2001 26. A meat pit pie was suspected to be involved in the Spanish *C. baratii* outbreak, but no leftover was available for analysis . In the other outbreaks of C. baratii botulism including that identified in France in 2014, all the food samples which were investigated, were negative for the detection of C. baratii and/or BoNT/F 7 . In the outbreak reported here, *C. baratii* was identified in two meat samples from the batch which had been used for the Bolognese sauce preparation, but they did not contain detectable BoNT level (Table 1). The Bolognese sauce was prepared at least 24 h in advance and was left several hours at room temperature during the hot summer period, thus constituting favourable conditions for *C. baratii* growth and toxin production. No leftover of the Bolognese sauce was available for analysis, but it was the likely source of contamination. Indeed, the fact that the strains isolated from the patients and meat samples were genetically identical supports that the meat used for the Bolognese sauce was responsible for this botulism outbreak. The minced beef was prepared in large batches and stored at -20°C by an industrial company. *C. baratii* was not detected in 26 frozen meat samples from the company which were related to the batch used by the restaurant. The source and mode of contamination of the beef remain unknown. This is the second outbreak of C. baratii botulism in France, 10 months after the previous one. The two outbreaks were unrelated based on the genetic differences between the *C. baratii* strains.

## Corresponding Author

Michel-Robert Popoff: michel-robert.popoff@pasteur.fr

## Competing Interests Statement

The authors have declared that no competing interests exist.

## Data Availability Statement

The whole-genome shotgun projects of the C. baratii strains 796-15, 693-15, 694-15 and 695-15 have been deposited at DDBJ/ENA/GenBank under the accession LUSO00000000, MJHL00000000, MJHM00000000, MJHN00000000, respectively. The versions described in this paper are version LUSO01000000, MJHL01000000, MJHM01000000 and MJHN01000000.

## References

[1] Popoff MR, Mazuet C, Poulain B. Botulism and Tetanus. The Prokaryotes: Human Microbiology. Berlin Heidelberg: Springer-Verlag; 2013. pp. 247-290.

[2] Smith TJ, Hill KK, Xie G, Foley BT, Williamson CH, Foster JT, et al. Genomic sequences of six botulinum neurotoxin-producing strains representing three clostridial species illustrate the mobility and diversity of botulinum neurotoxin genes. Infect Genet Evol. 2015;30:102-13. 10.1016/j.meegid.2014.12.002 PMC5459376 25489752

[3] Carlier JP, Espié E, Popoff MR. Le botulisme en France, 2003-2006. Bull Epidemiol Hebdo. 2007;31-32:281-4.

[4] Mazuet C, Bouvet P, King LA, Popoff MR. Le botulisme humain en France, 2007-2009. BEH. 2011;6:49-53.

[5] Mazuet C, King LA, Bouvet P, Legeay C, Sautereau J, Popoff MR. Le botulisme humain en France, 2010-2012. BEH. 2014;6:106-14.

[6] Moller V, Scheibel I. Preliminary report on the isolation of an apparently new type of Clostridium botulinum. Acta Pathol Microbiol Scand. 1960;48:80. 10.1111/j.1699-0463.1960.tb04741.x 14423425

[7] Gupta A, Sumner CJ, Castor M, Maslanka S, Sobel J. Adult botulism type F in the United States, 1981-2002. Neurology. 2005;65(11):1694-700. 10.1212/01.wnl.0000187127.92446.4c 16344510

[8] Hall JD, McCroskey LM, Pincomb BJ, Hatheway CL. Isolation of an organism resembling Clostridium baratii which produces type F botulinal toxin from an infant with botulism. J Clin Microbiol. 1985;21:654-5. 10.1128/jcm.21.4.654-655.1985 PMC271744 3988908

[9] Hill KK, Smith TJ. Genetic diversity within Clostridium botulinum serotypes, botulinum neurotoxin gene clusters and toxin subtypes. Curr Top Microbiol Immunol. 2013;364(doi):1-20. 10.1007/978-3-642-33570-9_1 23239346

[10] Dabritz HA, Hill KK, Barash JR, Ticknor LO, Helma CH, Dover N, et al. Molecular Epidemiology of Infant Botulism in California and Elsewhere, 1976-2010. J Infect Dis. 2014;210:1711-22. 10.1093/infdis/jiu331 24924163

[11] Fox CK, Keet CA, Strober JB. Recent advances in infant botulism. Pediatr Neurol. 2005;32(3):149-54. 10.1016/j.pediatrneurol.2004.10.001 15730893

[12] Moodley A, Quinlisk P, Garvey A, Kalas N, Barash JR, Khouri JM. Notes from the field: infant botulism caused by Clostridium baratii type F - Iowa, 2013. MMWR Morb Mortal Wkly Rep. 2015;64(14):400. PMC5779545 25879901

[13] Keet CA, Fox CK, Margeta M, Marco E, Shane AL, Dearmond SJ, et al. Infant botulism, type F, presenting at 54 hours of life. Pediatr Neurol. 2005;32(3):193-6. 10.1016/j.pediatrneurol.2004.09.003 15730901

[14] Hannett GE, Schaffzin JK, Davis SW, Fage MP, Schoonmaker-Bopp D, Dumas NB, et al. Two cases of adult botulism caused by botulinum neurotoxin producing Clostridium baratii. Anaerobe. 2014;30C:178-180. 10.1016/j.anaerobe.2014.10.005 25463969

[15] Trehard H, Poujol I, Mazuet C, Blanc Q, Gillet Y, Rossignol F, et al. A cluster of three cases of botulism due to Clostridium baratii type F, France, August 2015. Euro Surveill. 2016;21(4). 10.2807/1560-7917.ES.2016.21.4.30117 26848055

[16] Mazuet C, Sautereau J, Legeay C, Bouchier C, Bouvet P, Jourdan da Silva N, et al. Characterization of the first Clostridium baratii strain responsible for an outbreak of botulism type F in France. Clin Microbiol Case Rep. 2015;1:1-4.

[17] Jousimies-Somer HR, Summanen P, Citron DM, Baron EJ, Wexler HM, Finegold SM. Anaerobic Bacteriology Manual. 6° ed. Belmont, California: Star Publishing Company; 2002.

[18] Vanhomwegen J, Berthet N, Mazuet C, Guigon G, Vallaeys T, Stamboliyska R, et al. Application of high-density DNA resequencing microarray for detection and characterization of botulinum neurotoxin-producing clostridia. PLoS One. 2013;8(6):e67510. 10.1371/journal.pone.0067510 PMC3688605 23818983

[19] Raphael BH, Choudoir MJ, Luquez C, Fernandez R, Maslanka SE. Sequence diversity of genes encoding botulinum neurotoxin type F. Appl Environ Microbiol. 2010;76(14):4805-12. 10.1128/AEM.03109-09 PMC2901728 20511432

[20] Dover N, Barash JR, Burke JN, Hill KK, Detter JC, Arnon SS. Arrangement of the Clostridium baratii F7 toxin gene cluster with identification of a sigma factor that recognizes the botulinum toxin gene cluster promoters. PLoS One. 2014;9(5):e97983. 10.1371/journal.pone.0097983 PMC4031146 24853378

[21] Kalb SR, Baudys J, Egan C, Smith TJ, Smith LA, Pirkle JL, et al. Different substrate recognition requirements for cleavage of synaptobrevin-2 by Clostridium baratii and Clostridium botulinum type F neurotoxins. Appl Environ Microbiol. 2011;77(4):1301-8. 10.1128/AEM.01662-10 PMC3067225 21169446

[22] Carver TJ, Rutherford KM, Berriman M, Rajandream MA, Barrell BG, Parkhill J. ACT: the Artemis Comparison Tool. Bioinformatics. 2005;21(16):3422-3. 10.1093/bioinformatics/bti553 15976072

[23] Guindon S, Dufayard JF, Lefort V, Anisimova M, Hordijk W, Gascuel O. New algorithms and methods to estimate maximum-likelihood phylogenies: assessing the performance of PhyML 3.0. Syst Biol. 2010;59(3):307-21. 10.1093/sysbio/syq010 20525638

[24] Guindon S, Gascuel O. A simple, fast, and accurate algorithm to estimate large phylogenies by maximum likelihood. Syst Biol. 2003;52(5):696-704. 10.1080/10635150390235520 14530136

[25] Barash JR, Tang TW, Arnon SS. First case of infant botulism caused by Clostridium baratii type F in California. J Clin Microbiol. 2005;43(8):4280-2. 10.1128/JCM.43.8.4280-4282.2005 PMC1233924 16082001

[26] Harvey SM, Sturgeon J, Dassey DE. Botulism due to Clostridium baratii type F toxin. J Clin Microbiol. 2002;40(6):2260-2. 10.1128/JCM.40.6.2260-2262.2002 PMC130751 12037104

[27] McCroskey LM, Hatheway CL, Woodruff BA, Greenberg JA, Jurgenson P. Type F botulism due to neurotoxigenic Clostridium baratii from an unknown source in an adult. J Clin Microbiol. 1991;29(11):2618-20. 10.1128/jcm.29.11.2618-2620.1991 PMC270386 1774272

[28] Paisley JW, Lauer BA, Arnon SS. A second case of infant botulism type F caused by Clostridium baratii. Pediatr Infect Dis J. 1995;14(10):912-4. 8584326

[29] Hill KK, Xie G, Foley BT, Smith TJ, Munk AC, Bruce D, et al. Recombination and insertion events involving the botulinum neurotoxin complex genes in Clostridium botulinum types A, B, E and F and Clostridium butyricum type E strains. BMC Biol. 2009;7:66. 10.1186/1741-7007-7-66 PMC2764570 19804621

[30] Hill KK, Xie G, Foley BT, Smith TJ. Genetic diversity within the botulinum neurotoxin-producing bacteria and their neurotoxins. Toxicon. 2015;107:2-8ki. 10.1016/j.toxicon.2015.09.011 26368006

[31] Smith TJ, Hill KK, Raphael BH. Historical and current perspectives on Clostridium botulinum diversity. Res Microbiol. 2015;166(4):290-302. 10.1016/j.resmic.2014.09.007 PMC11302483 25312020

[32] Treangen TJ, Ondov BD, Koren S, Phillippy AM. The Harvest suite for rapid core-genome alignment and visualization of thousands of intraspecific microbial genomes. Genome Biol 2014; 15 (11):524. 10.1186/s13059-014-0524-x PMC4262987 25410596

[33] Barash JR, Arnon SS. Dual toxin-producing strain of Clostridium botulinum type Bf isolated from a California patient with infant botulism. J Clin Microbiol 2004; 42 (4):1713-1715. 10.1128/JCM.42.4.1713-1715.2004 PMC387584 15071029

[34] Castor C, Mazuet C, Saint-Leger M, Vygen S, Coutureau J, Durand M, et al. Cluster of two cases of botulism due to Clostridium baratii type F in France, November 2014. Euro Surveill 2015; 20 (6):1-3. 10.2807/1560-7917.es2015.20.6.21031 25695475

[35] Lafuente S, Nolla J, Valdezate S, Tortajada C, Vargas-Leguas H, Parron I, et al. Two simultaneous botulism outbreaks in Barcelona: Clostridium baratii and Clostridium botulinum. Epidemiol Infect 2012; 19:1-3. 10.1017/S0950268812002592 PMC9151435 23158693

